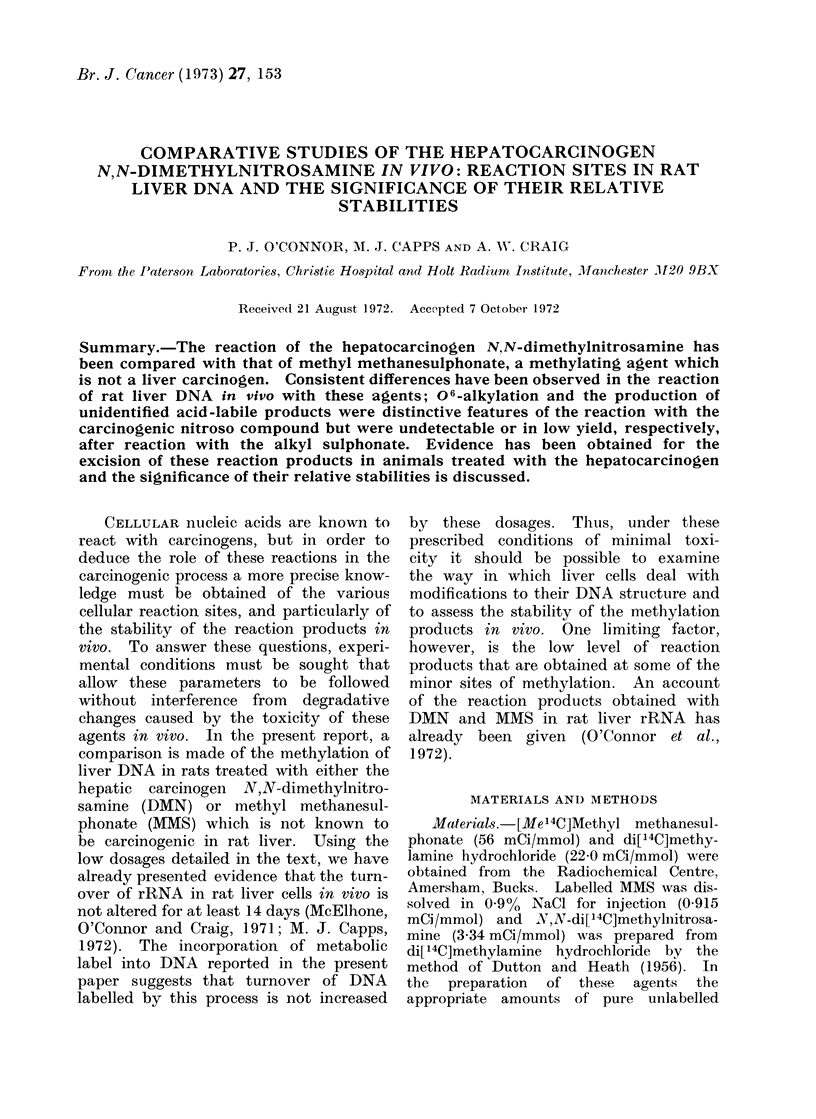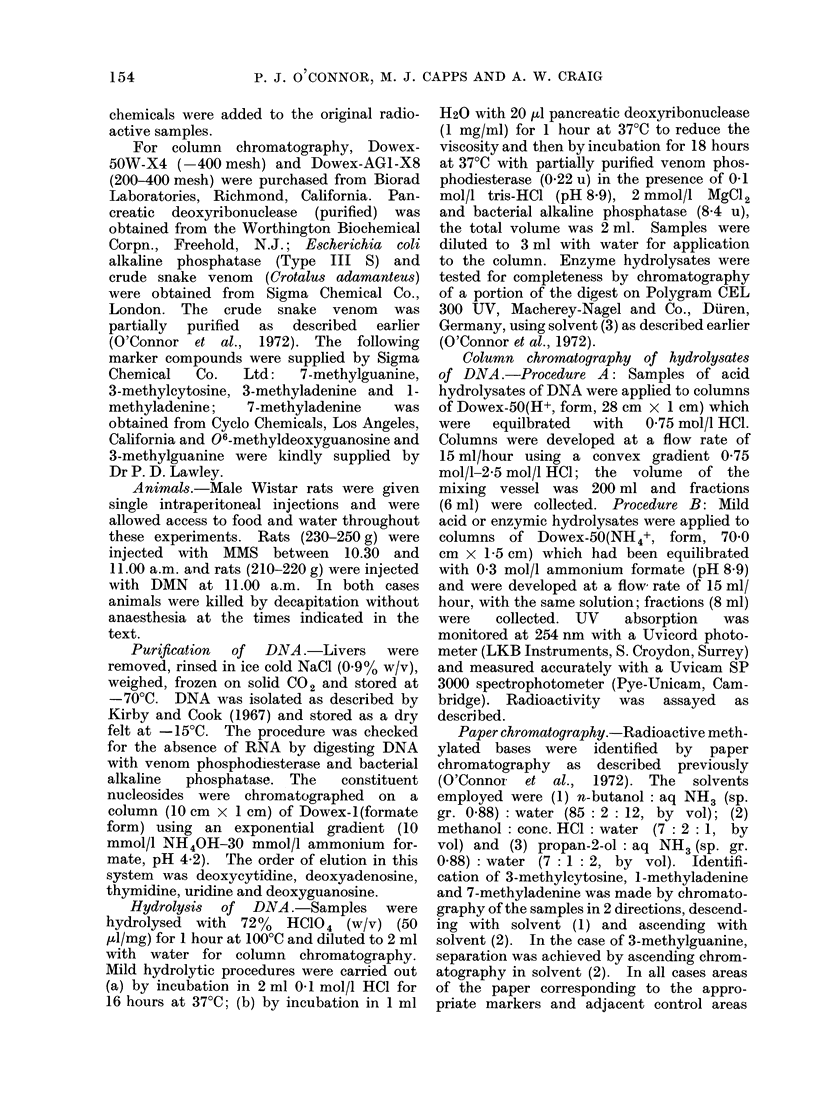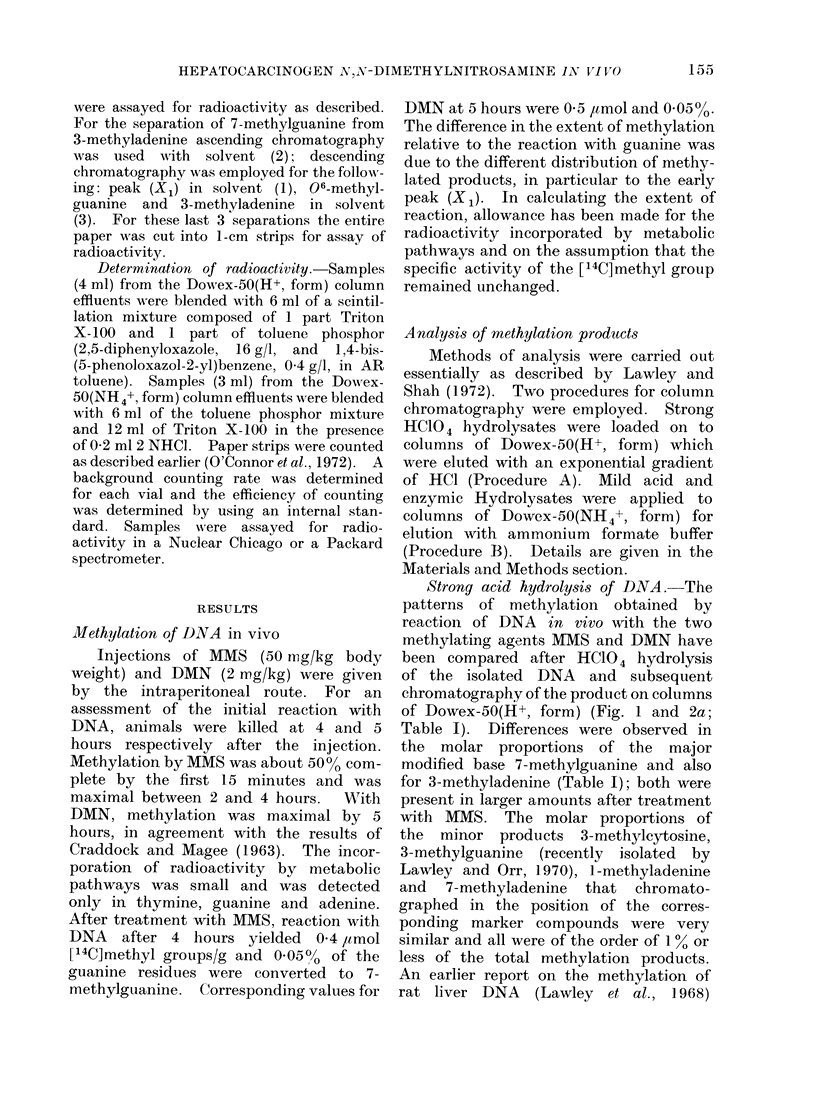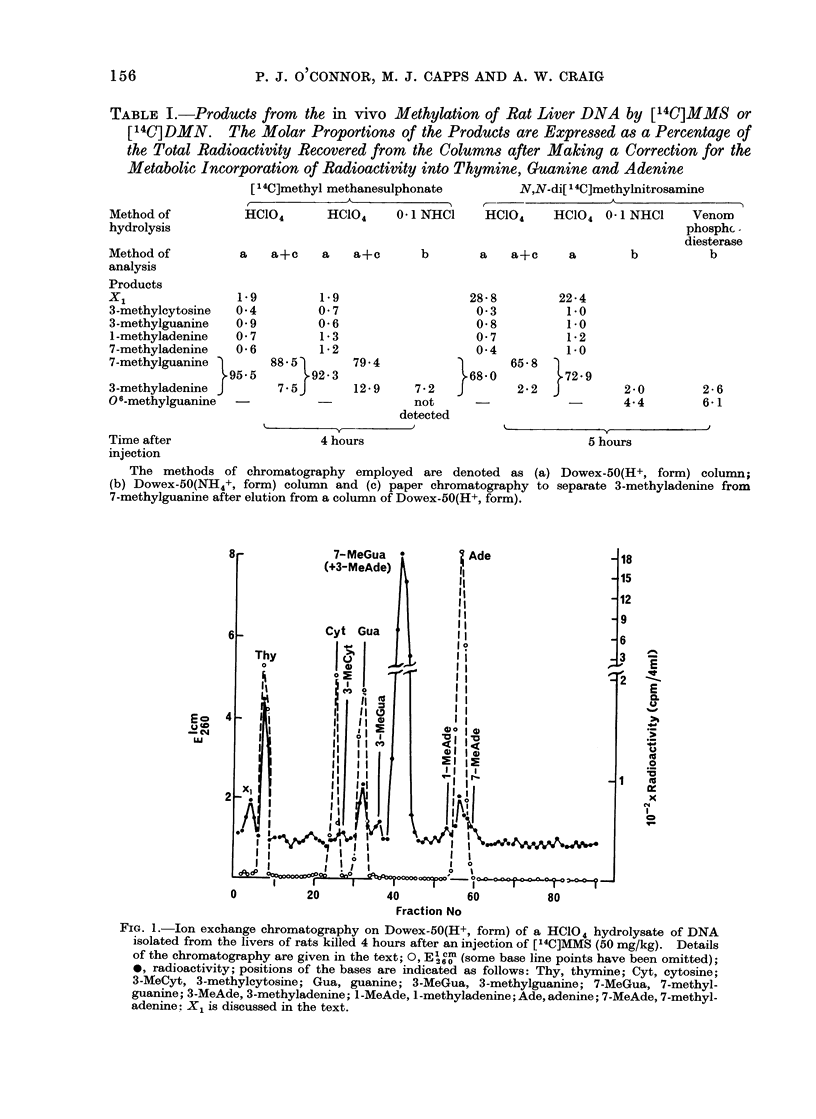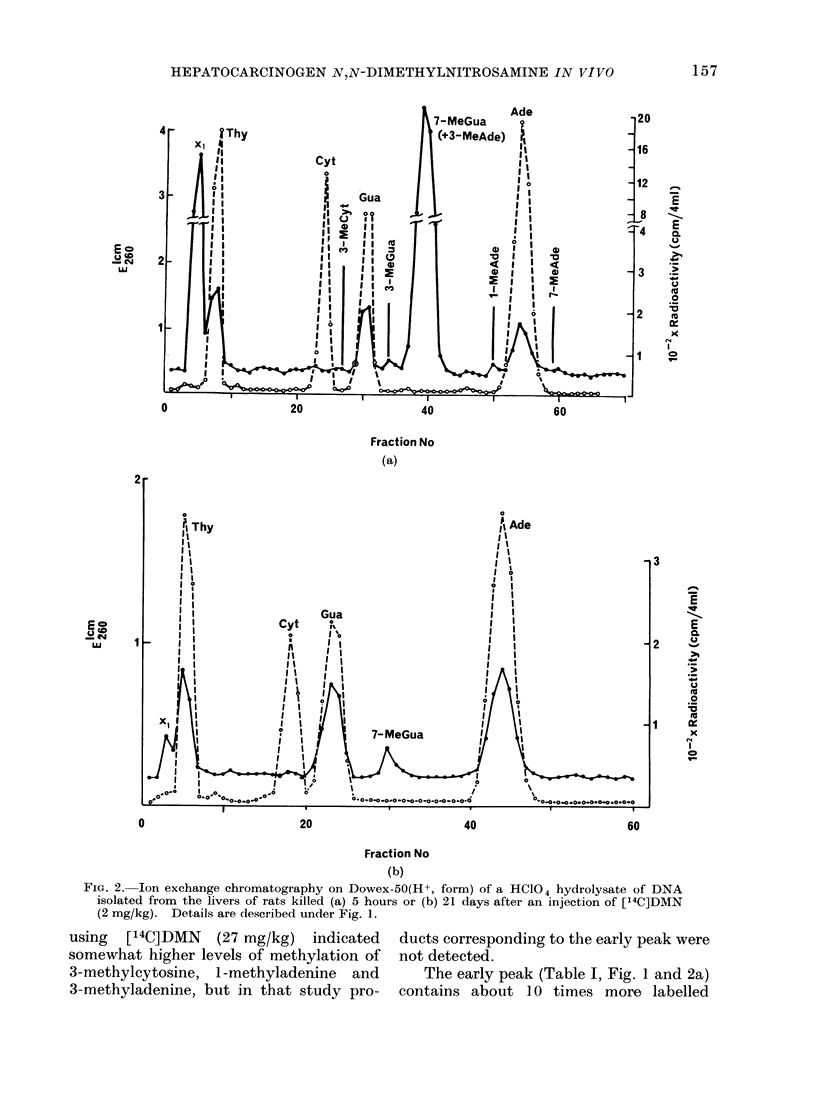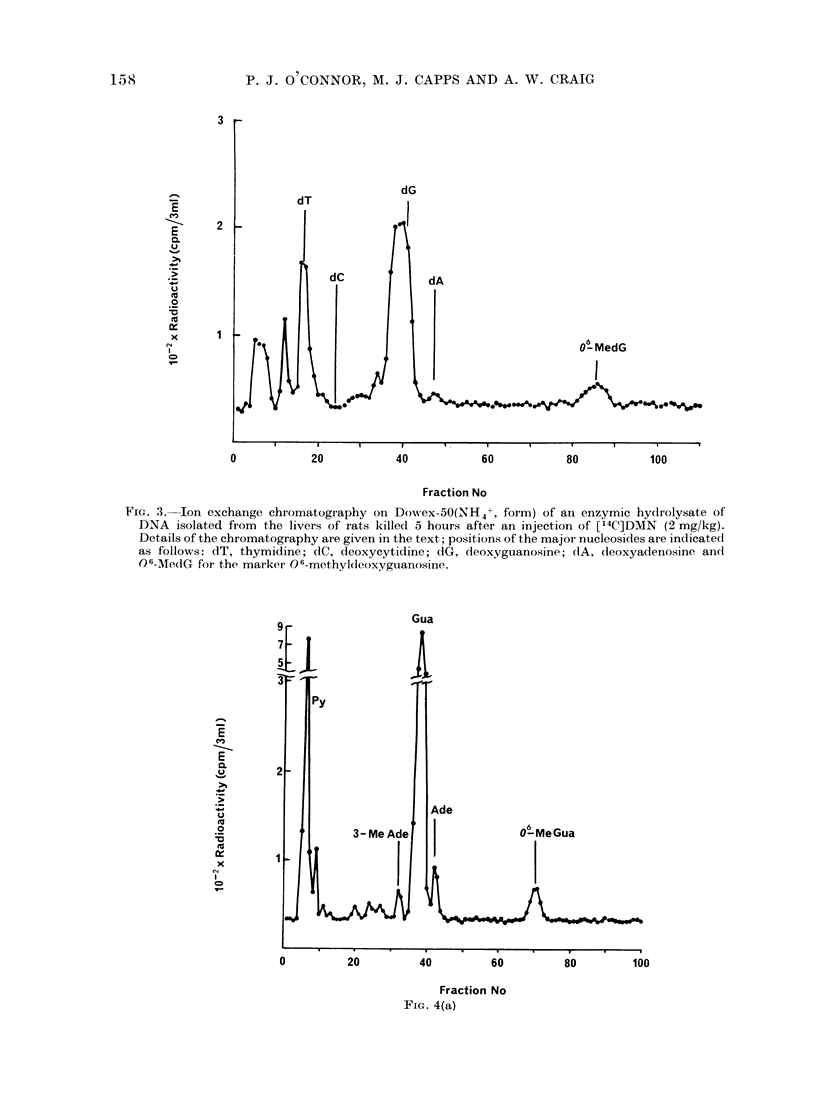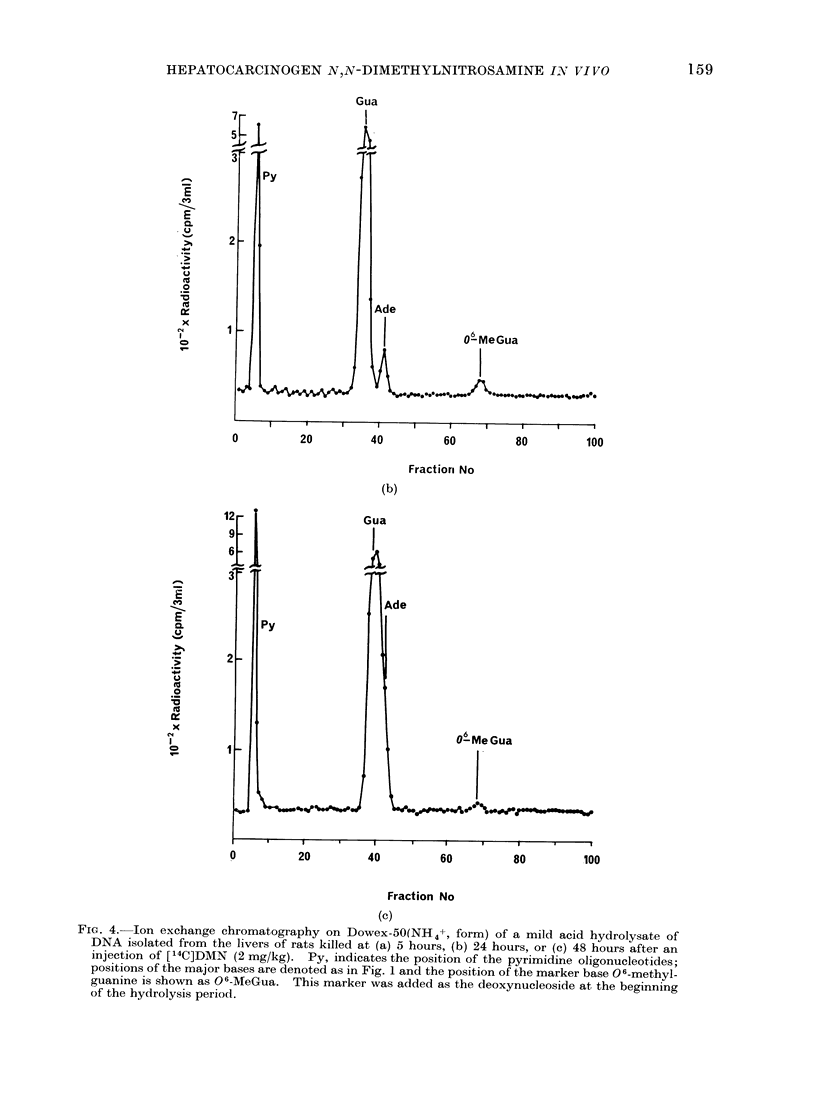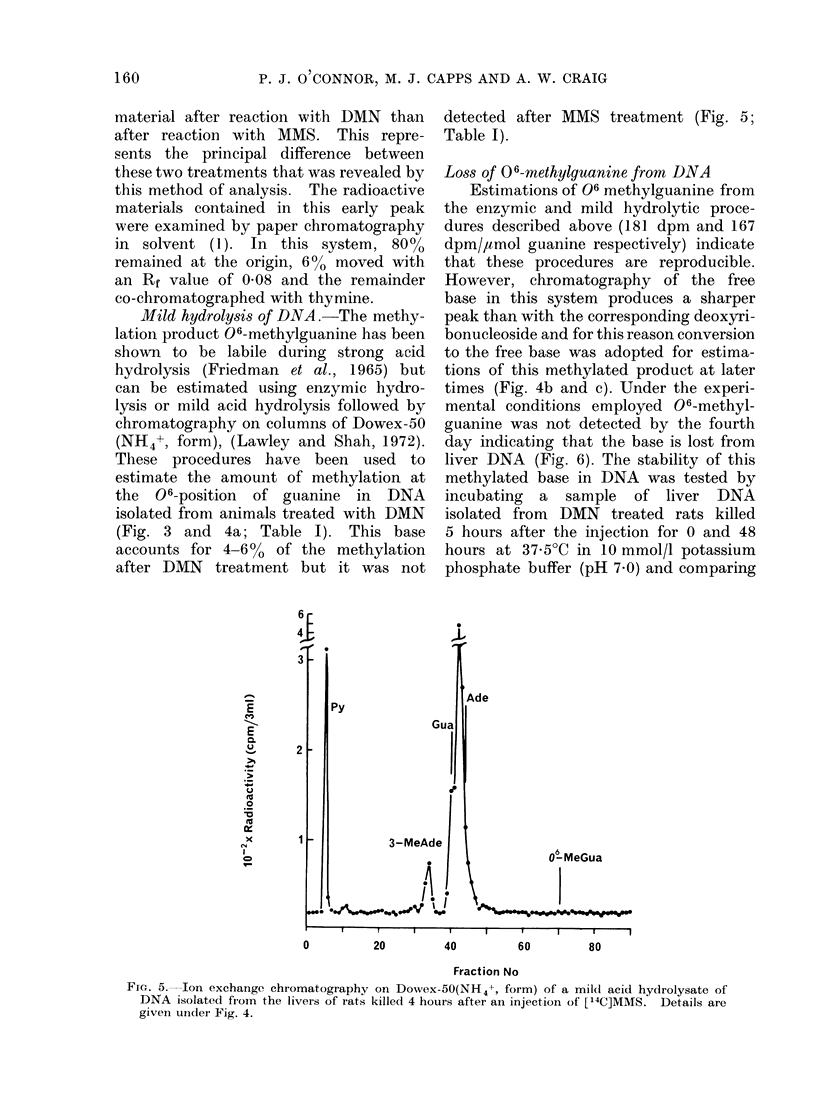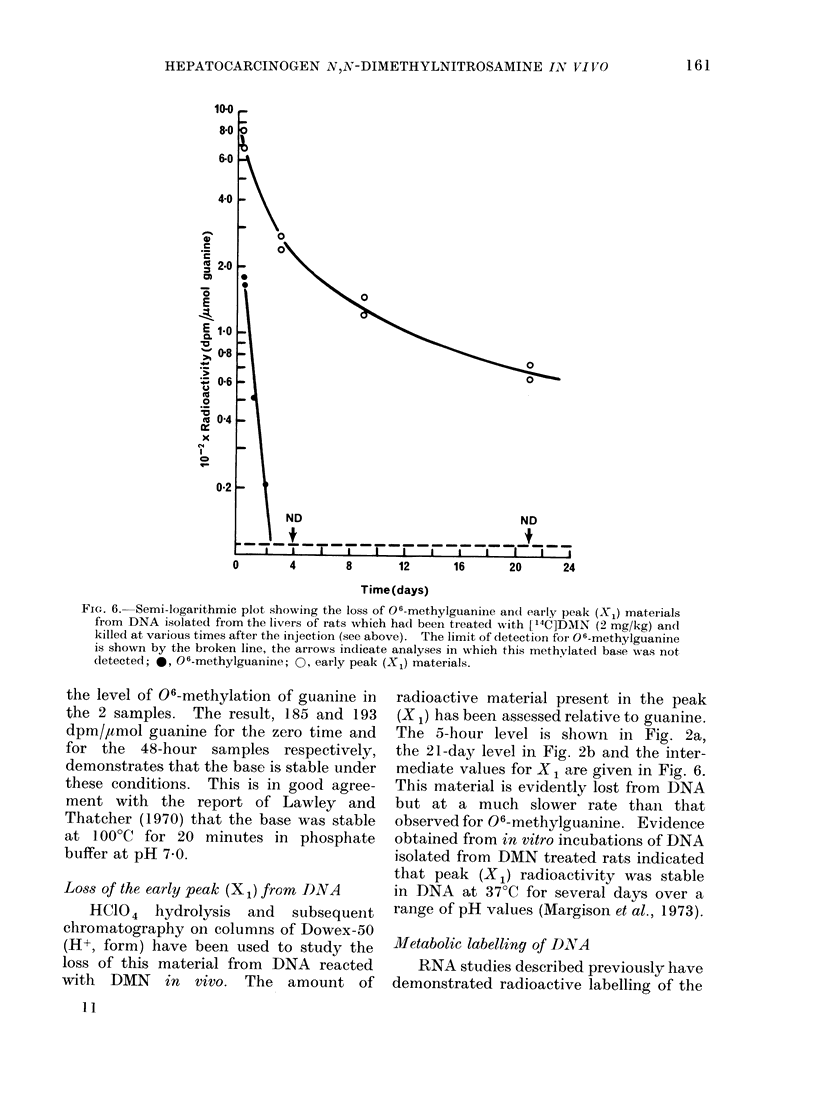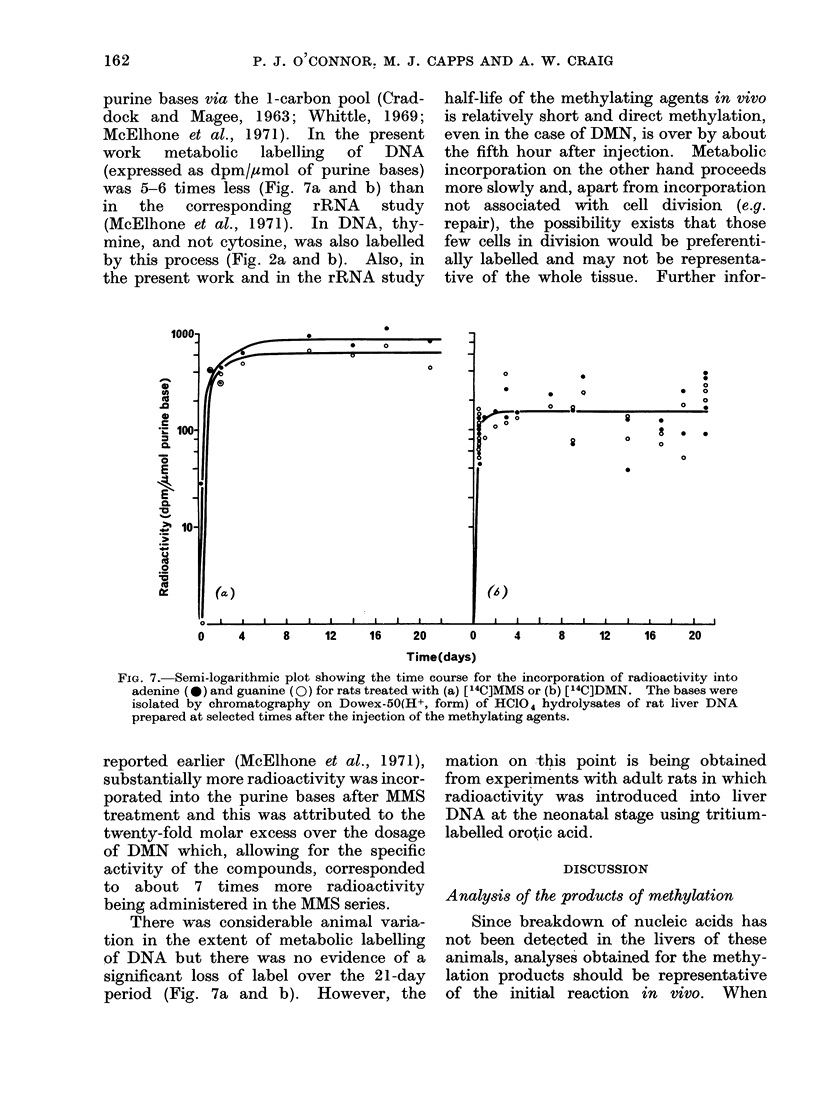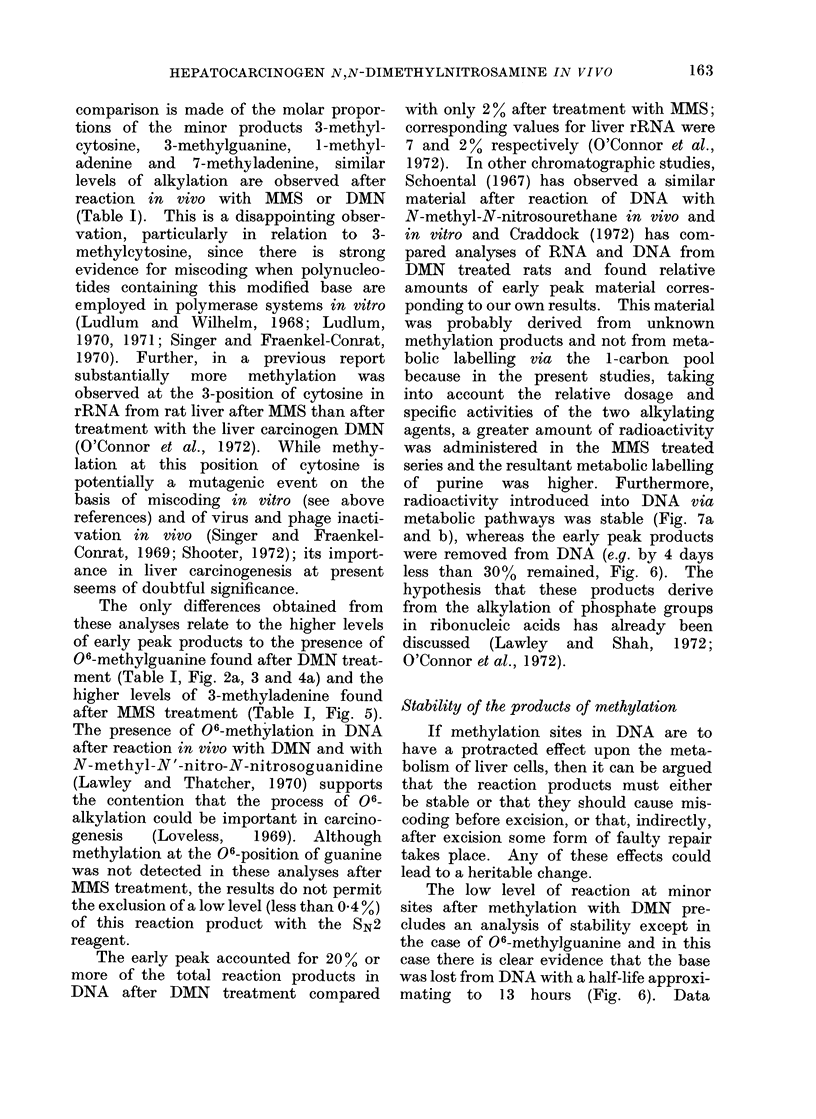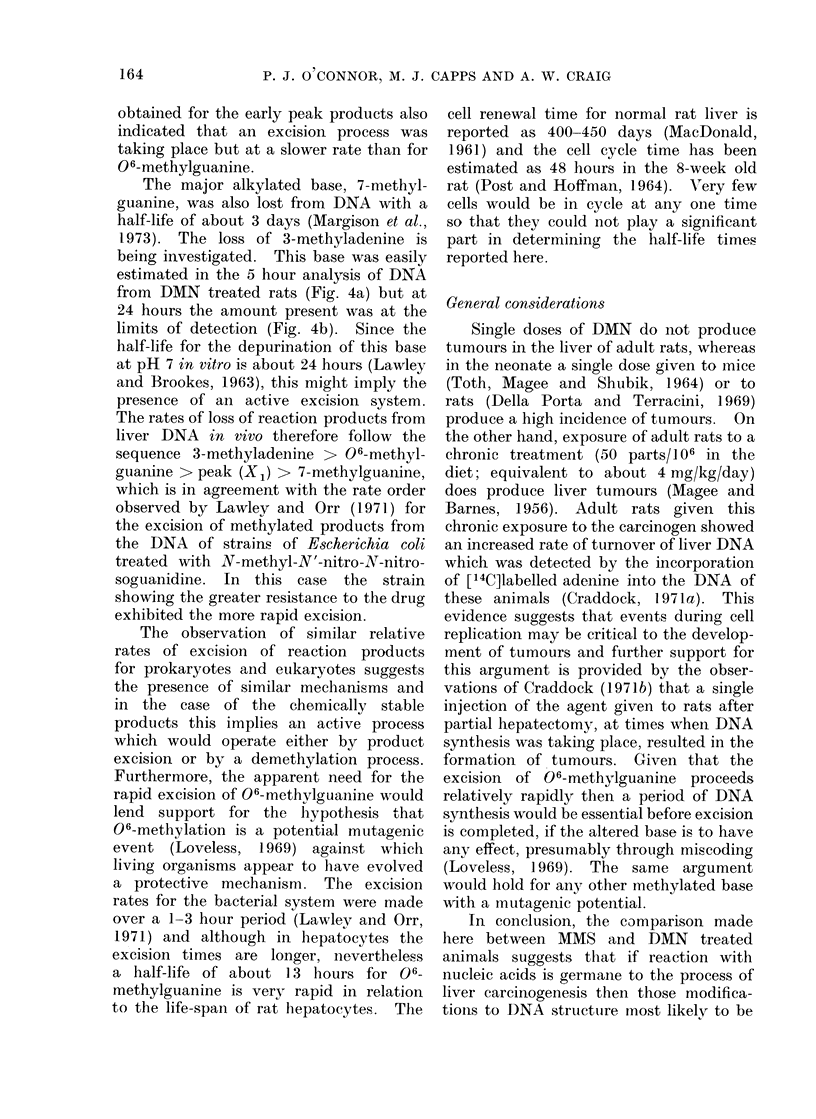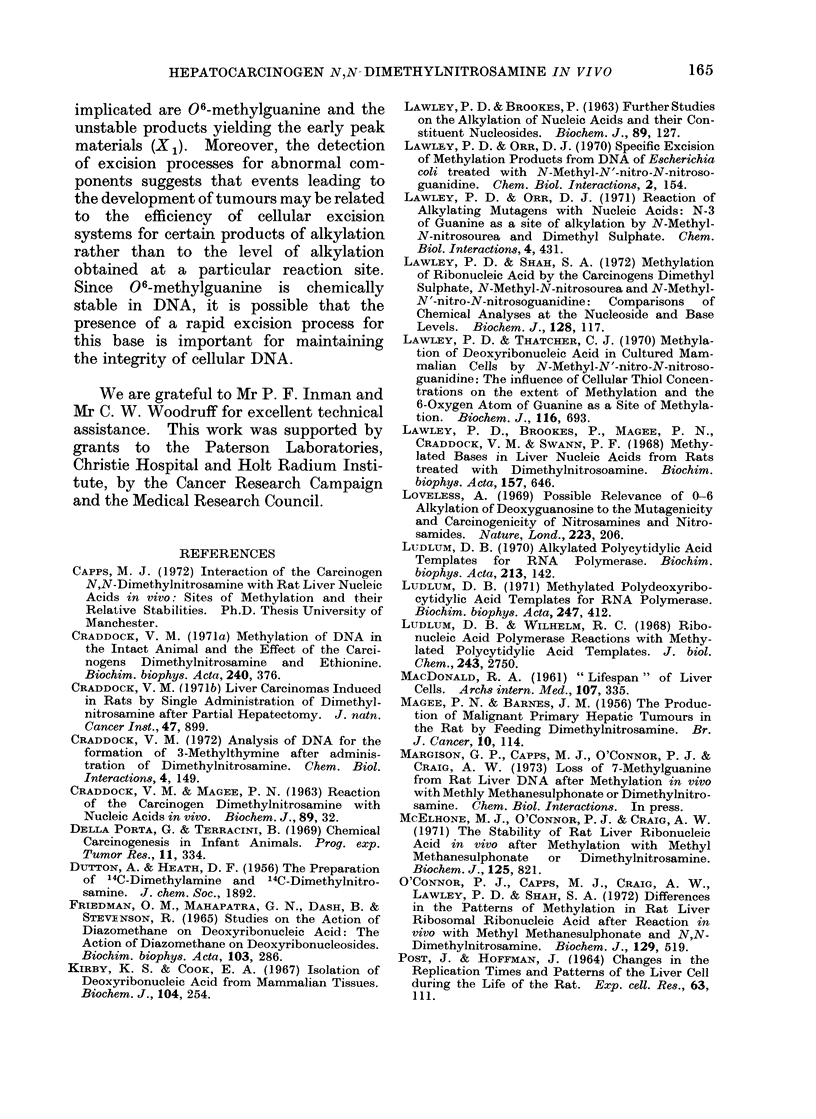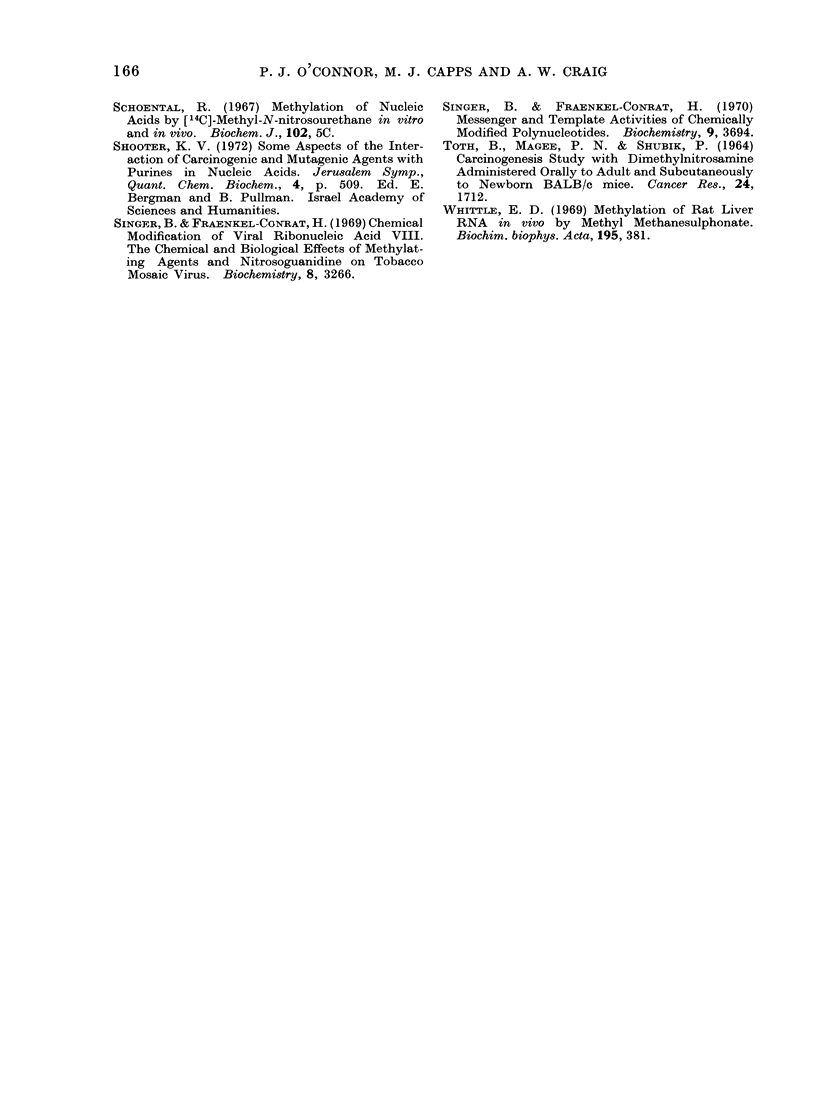# Comparative Studies of the Hepatocarcinogen N,N-Dimethylnitrosamine in vivo: Reaction Sites in Rat Liver DNA and the Significance of their Relative Stabilities

**DOI:** 10.1038/bjc.1973.19

**Published:** 1973-02

**Authors:** P. J. O'Connor, M. J. Capps, A. W. Craig

## Abstract

The reaction of the hepatocarcinogen *N,N*-dimethylnitrosamine has been compared with that of methyl methanesulphonate, a methylating agent which is not a liver carcinogen. Consistent differences have been observed in the reaction of rat liver DNA *in vivo* with these agents; *O*^6^-alkylation and the production of unidentified acid-labile products were distinctive features of the reaction with the carcinogenic nitroso compound but were undetectable or in low yield, respectively, after reaction with the alkyl sulphonate. Evidence has been obtained for the excision of these reaction products in animals treated with the hepatocarcinogen and the significance of their relative stabilities is discussed.


*Br. J. Cancer* (1973) **27**, 153

### COMPARATIVE STUDIES OF THE HEPATOCARCINOGEN *N,N*-DIMETHYLNITROSAMINE *IN VIVO*: REACTION SITES IN RAT LIVER DNA AND THE SIGNIFICANCE OF THEIR RELATIVE STABILITIES

P. J. O'CONNOR, M. J. CAPPS AND A. W. CRAIG

*From the Paterson Laboratories, Christie Hospital and Holt Radium Institute, Manchester M20 9BX*

Received 21 August 1972. Accepted 7 October 1972

**Summary.—The reaction of the hepatocarcinogen *N,N*-dimethylnitrosamine has been compared with that of methyl methanesulphonate, a methylating agent which is not a liver carcinogen. Consistent differences have been observed in the reaction of rat liver DNA *in vivo* with these agents; $O^6$-alkylation and the production of unidentified acid-labile products were distinctive features of the reaction with the carcinogenic nitroso compound but were undetectable or in low yield, respectively, after reaction with the alkyl sulphonate. Evidence has been obtained for the excision of these reaction products in animals treated with the hepatocarcinogen and the significance of their relative stabilities is discussed.**

CELLULAR nucleic acids are known to react with carcinogens, but in order to deduce the role of these reactions in the carcinogenic process a more precise knowledge must be obtained of the various cellular reaction sites, and particularly of the stability of the reaction products *in vivo*. To answer these questions, experimental conditions must be sought that allow these parameters to be followed without interference from degradative changes caused by the toxicity of these agents *in vivo*. In the present report, a comparison is made of the methylation of liver DNA in rats treated with either the hepatic carcinogen *N,N*-dimethylnitrosamine (DMN) or methyl methanesulphonate (MMS) which is not known to be carcinogenic in rat liver. Using the low dosages detailed in the text, we have already presented evidence that the turnover of rRNA in rat liver cells *in vivo* is not altered for at least 14 days (McElhone, O'Connor and Craig, 1971; M. J. Capps, 1972). The incorporation of metabolic label into DNA reported in the present paper suggests that turnover of DNA labelled by this process is not increased by these dosages. Thus, under these prescribed conditions of minimal toxicity it should be possible to examine the way in which liver cells deal with modifications to their DNA structure and to assess the stability of the methylation products *in vivo*. One limiting factor, however, is the low level of reaction products that are obtained at some of the minor sites of methylation. An account of the reaction products obtained with DMN and MMS in rat liver rRNA has already been given (O'Connor *et al.*, 1972).

### MATERIALS AND METHODS

*Materials.*—[$Me^{14}$C]Methyl methanesulphonate (56 mCi/mmol) and di[$^{14}$C]methylamine hydrochloride (22·0 mCi/mmol) were obtained from the Radiochemical Centre, Amersham, Bucks. Labelled MMS was dissolved in 0·9% NaCl for injection (0·915 mCi/mmol) and *N,N*-di[$^{14}$C]methylnitrosamine (3·34 mCi/mmol) was prepared from di[$^{14}$C]methylamine hydrochloride by the method of Dutton and Heath (1956). In the preparation of these agents the appropriate amounts of pure unlabelled chemicals were added to the original radioactive samples.

For column chromatography, Dowex-50W-X4 (—400 mesh) and Dowex-AG1-X8 (200–400 mesh) were purchased from Biorad Laboratories, Richmond, California. Pancreatic deoxyribonuclease (purified) was obtained from the Worthington Biochemical Corpn., Freehold, N.J.; *Escherichia coli* alkaline phosphatase (Type III S) and crude snake venom (*Crotalus adamanteus*) were obtained from Sigma Chemical Co., London. The crude snake venom was partially purified as described earlier (O'Connor *et al.*, 1972). The following marker compounds were supplied by Sigma Chemical Co. Ltd: 7-methylguanine, 3-methylcytosine, 3-methyladenine and 1-methyladenine; 7-methyladenine was obtained from Cyclo Chemicals, Los Angeles, California and $O^6$-methyldeoxyguanosine and 3-methylguanine were kindly supplied by Dr P. D. Lawley.

*Animals.*—Male Wistar rats were given single intraperitoneal injections and were allowed access to food and water throughout these experiments. Rats (230–250 g) were injected with MMS between 10.30 and 11.00 a.m. and rats (210–220 g) were injected with DMN at 11.00 a.m. In both cases animals were killed by decapitation without anaesthesia at the times indicated in the text.

*Purification of DNA.*—Livers were removed, rinsed in ice cold NaCl (0·9% w/v), weighed, frozen on solid $CO_2$ and stored at —70°C. DNA was isolated as described by Kirby and Cook (1967) and stored as a dry felt at —15°C. The procedure was checked for the absence of RNA by digesting DNA with venom phosphodiesterase and bacterial alkaline phosphatase. The constituent nucleosides were chromatographed on a column (10 cm × 1 cm) of Dowex-1(formate form) using an exponential gradient (10 mmol/l $NH_4OH$–30 mmol/l ammonium formate, pH 4·2). The order of elution in this system was deoxycytidine, deoxyadenosine, thymidine, uridine and deoxyguanosine.

*Hydrolysis of DNA.*—Samples were hydrolysed with 72% $HClO_4$ (w/v) (50 μl/mg) for 1 hour at 100°C and diluted to 2 ml with water for column chromatography. Mild hydrolytic procedures were carried out (a) by incubation in 2 ml 0·1 mol/l HCl for 16 hours at 37°C; (b) by incubation in 1 ml $H_2O$ with 20 μl pancreatic deoxyribonuclease (1 mg/ml) for 1 hour at 37°C to reduce the viscosity and then by incubation for 18 hours at 37°C with partially purified venom phosphodiesterase (0·22 u) in the presence of 0·1 mol/l tris-HCl (pH 8·9), 2 mmol/l $MgCl_2$ and bacterial alkaline phosphatase (8·4 u), the total volume was 2 ml. Samples were diluted to 3 ml with water for application to the column. Enzyme hydrolysates were tested for completeness by chromatography of a portion of the digest on Polygram CEL 300 UV, Macherey-Nagel and Co., Düren, Germany, using solvent (3) as described earlier (O'Connor *et al.*, 1972).

*Column chromatography of hydrolysates of DNA.—Procedure A*: Samples of acid hydrolysates of DNA were applied to columns of Dowex-50($H^+$, form, 28 cm × 1 cm) which were equilbrated with 0·75 mol/l HCl. Columns were developed at a flow rate of 15 ml/hour using a convex gradient 0·75 mol/l–2·5 mol/l HCl; the volume of the mixing vessel was 200 ml and fractions (6 ml) were collected. *Procedure B*: Mild acid or enzymic hydrolysates were applied to columns of Dowex-50($NH_4^+$, form, 70·0 cm × 1·5 cm) which had been equilibrated with 0·3 mol/l ammonium formate (pH 8·9) and were developed at a flow rate of 15 ml/hour, with the same solution; fractions (8 ml) were collected. UV absorption was monitored at 254 nm with a Uvicord photometer (LKB Instruments, S. Croydon, Surrey) and measured accurately with a Uvicam SP 3000 spectrophotometer (Pye-Unicam, Cambridge). Radioactivity was assayed as described.

*Paper chromatography.*—Radioactive methylated bases were identified by paper chromatography as described previously (O'Connor *et al.*, 1972). The solvents employed were (1) *n*-butanol : aq $NH_3$ (sp. gr. 0·88) : water (85 : 2 : 12, by vol); (2) methanol : conc. HCl : water (7 : 2 : 1, by vol) and (3) propan-2-ol : aq $NH_3$ (sp. gr. 0·88) : water (7 : 1 : 2, by vol). Identification of 3-methylcytosine, 1-methyladenine and 7-methyladenine was made by chromatography of the samples in 2 directions, descending with solvent (1) and ascending with solvent (2). In the case of 3-methylguanine, separation was achieved by ascending chromatography in solvent (2). In all cases areas of the paper corresponding to the appropriate markers and adjacent control areas were assayed for radioactivity as described. For the separation of 7-methylguanine from 3-methyladenine ascending chromatography was used with solvent (2); descending chromatography was employed for the following: peak ($X_1$) in solvent (1), $O^6$-methylguanine and 3-methyladenine in solvent (3). For these last 3 separations the entire paper was cut into 1-cm strips for assay of radioactivity.

*Determination of radioactivity.*—Samples (4 ml) from the Dowex-50($H^+$, form) column effluents were blended with 6 ml of a scintillation mixture composed of 1 part Triton X-100 and 1 part of toluene phosphor (2,5-diphenyloxazole, 16 g/l, and 1,4-bis-(5-phenoloxazol-2-yl)benzene, 0·4 g/l, in AR toluene). Samples (3 ml) from the Dowex-50($NH_4^+$, form) column effluents were blended with 6 ml of the toluene phosphor mixture and 12 ml of Triton X-100 in the presence of 0·2 ml 2 NHCl. Paper strips were counted as described earlier (O'Connor *et al.*, 1972). A background counting rate was determined for each vial and the efficiency of counting was determined by using an internal standard. Samples were assayed for radioactivity in a Nuclear Chicago or a Packard spectrometer.

### RESULTS

### *Methylation of DNA* in vivo

Injections of MMS (50 mg/kg body weight) and DMN (2 mg/kg) were given by the intraperitoneal route. For an assessment of the initial reaction with DNA, animals were killed at 4 and 5 hours respectively after the injection. Methylation by MMS was about 50% complete by the first 15 minutes and was maximal between 2 and 4 hours. With DMN, methylation was maximal by 5 hours, in agreement with the results of Craddock and Magee (1963). The incorporation of radioactivity by metabolic pathways was small and was detected only in thymine, guanine and adenine. After treatment with MMS, reaction with DNA after 4 hours yielded 0·4 μmol [$^{14}$C]methyl groups/g and 0·05% of the guanine residues were converted to 7-methylguanine. Corresponding values for DMN at 5 hours were 0·5 μmol and 0·05%. The difference in the extent of methylation relative to the reaction with guanine was due to the different distribution of methylated products, in particular to the early peak ($X_1$). In calculating the extent of reaction, allowance has been made for the radioactivity incorporated by metabolic pathways and on the assumption that the specific activity of the [$^{14}$C]methyl group remained unchanged.

### *Analysis of methylation products*

Methods of analysis were carried out essentially as described by Lawley and Shah (1972). Two procedures for column chromatography were employed. Strong $HClO_4$ hydrolysates were loaded on to columns of Dowex-50($H^+$, form) which were eluted with an exponential gradient of HCl (Procedure A). Mild acid and enzymic Hydrolysates were applied to columns of Dowex-50($NH_4^+$, form) for elution with ammonium formate buffer (Procedure B). Details are given in the Materials and Methods section.

*Strong acid hydrolysis of DNA.*—The patterns of methylation obtained by reaction of DNA *in vivo* with the two methylating agents MMS and DMN have been compared after $HClO_4$ hydrolysis of the isolated DNA and subsequent chromatography of the product on columns of Dowex-50($H^+$, form) (Fig. 1 and 2*a*; Table I). Differences were observed in the molar proportions of the major modified base 7-methylguanine and also for 3-methyladenine (Table I); both were present in larger amounts after treatment with MMS. The molar proportions of the minor products 3-methylcytosine, 3-methylguanine (recently isolated by Lawley and Orr, 1970), 1-methyladenine and 7-methyladenine that chromatographed in the position of the corresponding marker compounds were very similar and all were of the order of 1% or less of the total methylation products. An earlier report on the methylation of rat liver DNA (Lawley *et al.*, 1968)

TABLE I.—*Products from the* in vivo *Methylation of Rat Liver DNA by [$^{14}$C]MMS or [$^{14}$C]DMN. The Molar Proportions of the Products are Expressed as a Percentage of the Total Radioactivity Recovered from the Columns after Making a Correction for the Metabolic Incorporation of Radioactivity into Thymine, Guanine and Adenine*

| | [$^{14}$C]methyl methanesulphonate | | | | | *N,N*-di[$^{14}$C]methylnitrosamine | | | | | |
|---|---|---|---|---|---|---|---|---|---|---|---|
| Method of hydrolysis | $HClO_4$ | | $HClO_4$ | | 0·1 NHCl | $HClO_4$ | | $HClO_4$ | 0·1 NHCl | Venom phosphodiesterase | |
| Method of analysis | a | a+c | a | a+c | b | a | a+c | a | b | b | |
| Products | | | | | | | | | | | |
| $X_1$ | 1·9 | | 1·9 | | | 28·8 | | 22·4 | | | |
| 3-methylcytosine | 0·4 | | 0·7 | | | 0·3 | | 1·0 | | | |
| 3-methylguanine | 0·9 | | 0·6 | | | 0·8 | | 1·0 | | | |
| 1-methyladenine | 0·7 | | 1·3 | | | 0·7 | | 1·2 | | | |
| 7-methyladenine | 0·6 | | 1·2 | | | 0·4 | | 1·0 | | | |
| 7-methylguanine | 95·5 | 88·5 | 92·3 | 79·4 | | 68·0 | 65·8 | 72·9 | | | |
| 3-methyladenine | | 7·5 | | 12·9 | 7·2 | | 2·2 | | 2·0 | 2·6 | |
| $O^6$-methylguanine | — | | — | | not detected | — | | — | 4·4 | 6·1 | |
| Time after injection | 4 hours | | | | | 5 hours | | | | | |

The methods of chromatography employed are denoted as (a) Dowex-50($H^+$, form) column; (b) Dowex-50($NH_4^+$, form) column and (c) paper chromatography to separate 3-methyladenine from 7-methylguanine after elution from a column of Dowex-50($H^+$, form).

FIG. 1.—Ion exchange chromatography on Dowex-50($H^+$, form) of a $HClO_4$ hydrolysate of DNA isolated from the livers of rats killed 4 hours after an injection of [$^{14}$C]MMS (50 mg/kg). Details of the chromatography are given in the text; ○, $E^{1\,cm}_{260}$ (some base line points have been omitted); ●, radioactivity; positions of the bases are indicated as follows: Thy, thymine; Cyt, cytosine; 3-MeCyt, 3-methylcytosine; Gua, guanine; 3-MeGua, 3-methylguanine; 7-MeGua, 7-methylguanine; 3-MeAde, 3-methyladenine; 1-MeAde, 1-methyladenine; Ade, adenine; 7-MeAde, 7-methyladenine; $X_1$ is discussed in the text.

FIG. 2.—Ion exchange chromatography on Dowex-50($H^+$, form) of a $HClO_4$ hydrolysate of DNA isolated from the livers of rats killed (a) 5 hours or (b) 21 days after an injection of [$^{14}$C]DMN (2 mg/kg). Details are described under Fig. 1.

using [$^{14}$C]DMN (27 mg/kg) indicated somewhat higher levels of methylation of 3-methylcytosine, 1-methyladenine and 3-methyladenine, but in that study products corresponding to the early peak were not detected.

The early peak (Table I, Fig. 1 and 2a) contains about 10 times more labelled

Fig. 3.—Ion exchange chromatography on Dowex-50($NH_4^+$, form) of an enzymic hydrolysate of DNA isolated from the livers of rats killed 5 hours after an injection of [$^{14}$C]DMN (2 mg/kg). Details of the chromatography are given in the text; positions of the major nucleosides are indicated as follows: dT, thymidine; dC, deoxycytidine; dG, deoxyguanosine; dA, deoxyadenosine and $O^6$-MedG for the marker $O^6$-methyldeoxyguanosine.

Fig. 4(a)

(b)

(c)

FIG. 4.—Ion exchange chromatography on Dowex-50($NH_4^+$, form) of a mild acid hydrolysate of DNA isolated from the livers of rats killed at (a) 5 hours, (b) 24 hours, or (c) 48 hours after an injection of [$^{14}$C]DMN (2 mg/kg). Py, indicates the position of the pyrimidine oligonucleotides; positions of the major bases are denoted as in Fig. 1 and the position of the marker base $O^6$-methylguanine is shown as $O^6$-MeGua. This marker was added as the deoxynucleoside at the beginning of the hydrolysis period.

material after reaction with DMN than after reaction with MMS. This represents the principal difference between these two treatments that was revealed by this method of analysis. The radioactive materials contained in this early peak were examined by paper chromatography in solvent (1). In this system, 80% remained at the origin, 6% moved with an $R_f$ value of 0·08 and the remainder co-chromatographed with thymine.

*Mild hydrolysis of DNA*.—The methylation product $O^6$-methylguanine has been shown to be labile during strong acid hydrolysis (Friedman *et al.*, 1965) but can be estimated using enzymic hydrolysis or mild acid hydrolysis followed by chromatography on columns of Dowex-50 ($NH_4^+$, form), (Lawley and Shah, 1972). These procedures have been used to estimate the amount of methylation at the $O^6$-position of guanine in DNA isolated from animals treated with DMN (Fig. 3 and 4a; Table I). This base accounts for 4–6% of the methylation after DMN treatment but it was not detected after MMS treatment (Fig. 5; Table I).

*Loss of* O[6]*-methylguanine from DNA*

Estimations of $O^6$ methylguanine from the enzymic and mild hydrolytic procedures described above (181 dpm and 167 dpm/$\mu$mol guanine respectively) indicate that these procedures are reproducible. However, chromatography of the free base in this system produces a sharper peak than with the corresponding deoxyribonucleoside and for this reason conversion to the free base was adopted for estimations of this methylated product at later times (Fig. 4b and c). Under the experimental conditions employed $O^6$-methylguanine was not detected by the fourth day indicating that the base is lost from liver DNA (Fig. 6). The stability of this methylated base in DNA was tested by incubating a sample of liver DNA isolated from DMN treated rats killed 5 hours after the injection for 0 and 48 hours at 37·5°C in 10 mmol/l potassium phosphate buffer (pH 7·0) and comparing

FIG. 5.—Ion exchange chromatography on Dowex-50($NH_4^+$, form) of a mild acid hydrolysate of DNA isolated from the livers of rats killed 4 hours after an injection of [$^{14}$C]MMS. Details are given under Fig. 4.

FIG. 6.—Semi-logarithmic plot showing the loss of $O^6$-methylguanine and early peak ($X_1$) materials from DNA isolated from the livers of rats which had been treated with [$^{14}$C]DMN (2 mg/kg) and killed at various times after the injection (see above). The limit of detection for $O^6$-methylguanine is shown by the broken line, the arrows indicate analyses in which this methylated base was not detected; ●, $O^6$-methylguanine; ○, early peak ($X_1$) materials.

the level of $O^6$-methylation of guanine in the 2 samples. The result, 185 and 193 dpm/μmol guanine for the zero time and for the 48-hour samples respectively, demonstrates that the base is stable under these conditions. This is in good agreement with the report of Lawley and Thatcher (1970) that the base was stable at 100°C for 20 minutes in phosphate buffer at pH 7·0.

*Loss of the early peak* ($X_1$) *from DNA*

$HClO_4$ hydrolysis and subsequent chromatography on columns of Dowex-50 ($H^+$, form) have been used to study the loss of this material from DNA reacted with DMN *in vivo*. The amount of radioactive material present in the peak ($X_1$) has been assessed relative to guanine. The 5-hour level is shown in Fig. 2a, the 21-day level in Fig. 2b and the intermediate values for $X_1$ are given in Fig. 6. This material is evidently lost from DNA but at a much slower rate than that observed for $O^6$-methylguanine. Evidence obtained from *in vitro* incubations of DNA isolated from DMN treated rats indicated that peak ($X_1$) radioactivity was stable in DNA at 37°C for several days over a range of pH values (Margison *et al.*, 1973).

*Metabolic labelling of DNA*

RNA studies described previously have demonstrated radioactive labelling of the purine bases *via* the 1-carbon pool (Craddock and Magee, 1963; Whittle, 1969; McElhone *et al.*, 1971). In the present work metabolic labelling of DNA (expressed as dpm/$\mu$mol of purine bases) was 5–6 times less (Fig. 7a and b) than in the corresponding rRNA study (McElhone *et al.*, 1971). In DNA, thymine, and not cytosine, was also labelled by this process (Fig. 2a and b). Also, in the present work and in the rRNA study reported earlier (McElhone *et al.*, 1971), substantially more radioactivity was incorporated into the purine bases after MMS treatment and this was attributed to the twenty-fold molar excess over the dosage of DMN which, allowing for the specific activity of the compounds, corresponded to about 7 times more radioactivity being administered in the MMS series.

There was considerable animal variation in the extent of metabolic labelling of DNA but there was no evidence of a significant loss of label over the 21-day period (Fig. 7a and b). However, the half-life of the methylating agents *in vivo* is relatively short and direct methylation, even in the case of DMN, is over by about the fifth hour after injection. Metabolic incorporation on the other hand proceeds more slowly and, apart from incorporation not associated with cell division (*e.g.* repair), the possibility exists that those few cells in division would be preferentially labelled and may not be representative of the whole tissue. Further information on this point is being obtained from experiments with adult rats in which radioactivity was introduced into liver DNA at the neonatal stage using tritium-labelled orotic acid.

FIG. 7.—Semi-logarithmic plot showing the time course for the incorporation of radioactivity into adenine (●) and guanine (○) for rats treated with (a) [$^{14}$C]MMS or (b) [$^{14}$C]DMN. The bases were isolated by chromatography on Dowex-50($H^+$, form) of $HClO_4$ hydrolysates of rat liver DNA prepared at selected times after the injection of the methylating agents.

### DISCUSSION

### *Analysis of the products of methylation*

Since breakdown of nucleic acids has not been detected in the livers of these animals, analyses obtained for the methylation products should be representative of the initial reaction *in vivo*. When comparison is made of the molar proportions of the minor products 3-methylcytosine, 3-methylguanine, 1-methyladenine and 7-methyladenine, similar levels of alkylation are observed after reaction *in vivo* with MMS or DMN (Table I). This is a disappointing observation, particularly in relation to 3-methylcytosine, since there is strong evidence for miscoding when polynucleotides containing this modified base are employed in polymerase systems *in vitro* (Ludlum and Wilhelm, 1968; Ludlum, 1970, 1971; Singer and Fraenkel-Conrat, 1970). Further, in a previous report substantially more methylation was observed at the 3-position of cytosine in rRNA from rat liver after MMS than after treatment with the liver carcinogen DMN (O'Connor *et al.*, 1972). While methylation at this position of cytosine is potentially a mutagenic event on the basis of miscoding *in vitro* (see above references) and of virus and phage inactivation *in vivo* (Singer and Fraenkel-Conrat, 1969; Shooter, 1972); its importance in liver carcinogenesis at present seems of doubtful significance.

The only differences obtained from these analyses relate to the higher levels of early peak products to the presence of $O^6$-methylguanine found after DMN treatment (Table I, Fig. 2a, 3 and 4a) and the higher levels of 3-methyladenine found after MMS treatment (Table I, Fig. 5). The presence of $O^6$-methylation in DNA after reaction *in vivo* with DMN and with *N*-methyl-*N*′-nitro-*N*-nitrosoguanidine (Lawley and Thatcher, 1970) supports the contention that the process of $O^6$-alkylation could be important in carcinogenesis (Loveless, 1969). Although methylation at the $O^6$-position of guanine was not detected in these analyses after MMS treatment, the results do not permit the exclusion of a low level (less than 0·4%) of this reaction product with the $S_N2$ reagent.

The early peak accounted for 20% or more of the total reaction products in DNA after DMN treatment compared with only 2% after treatment with MMS; corresponding values for liver rRNA were 7 and 2% respectively (O'Connor *et al.*, 1972). In other chromatographic studies, Schoental (1967) has observed a similar material after reaction of DNA with *N*-methyl-*N*-nitrosourethane *in vivo* and *in vitro* and Craddock (1972) has compared analyses of RNA and DNA from DMN treated rats and found relative amounts of early peak material corresponding to our own results. This material was probably derived from unknown methylation products and not from metabolic labelling *via* the 1-carbon pool because in the present studies, taking into account the relative dosage and specific activities of the two alkylating agents, a greater amount of radioactivity was administered in the MMS treated series and the resultant metabolic labelling of purine was higher. Furthermore, radioactivity introduced into DNA *via* metabolic pathways was stable (Fig. 7a and b), whereas the early peak products were removed from DNA (*e.g.* by 4 days less than 30% remained, Fig. 6). The hypothesis that these products derive from the alkylation of phosphate groups in ribonucleic acids has already been discussed (Lawley and Shah, 1972; O'Connor *et al.*, 1972).

*Stability of the products of methylation*

If methylation sites in DNA are to have a protracted effect upon the metabolism of liver cells, then it can be argued that the reaction products must either be stable or that they should cause miscoding before excision, or that, indirectly, after excision some form of faulty repair takes place. Any of these effects could lead to a heritable change.

The low level of reaction at minor sites after methylation with DMN precludes an analysis of stability except in the case of $O^6$-methylguanine and in this case there is clear evidence that the base was lost from DNA with a half-life approximating to 13 hours (Fig. 6). Data obtained for the early peak products also indicated that an excision process was taking place but at a slower rate than for $O^6$-methylguanine.

The major alkylated base, 7-methylguanine, was also lost from DNA with a half-life of about 3 days (Margison *et al.*, 1973). The loss of 3-methyladenine is being investigated. This base was easily estimated in the 5 hour analysis of DNA from DMN treated rats (Fig. 4a) but at 24 hours the amount present was at the limits of detection (Fig. 4b). Since the half-life for the depurination of this base at pH 7 *in vitro* is about 24 hours (Lawley and Brookes, 1963), this might imply the presence of an active excision system. The rates of loss of reaction products from liver DNA *in vivo* therefore follow the sequence 3-methyladenine > $O^6$-methylguanine > peak ($X_1$) > 7-methylguanine, which is in agreement with the rate order observed by Lawley and Orr (1971) for the excision of methylated products from the DNA of strains of *Escherichia coli* treated with *N*-methyl-*N'*-nitro-*N*-nitrosoguanidine. In this case the strain showing the greater resistance to the drug exhibited the more rapid excision.

The observation of similar relative rates of excision of reaction products for prokaryotes and eukaryotes suggests the presence of similar mechanisms and in the case of the chemically stable products this implies an active process which would operate either by product excision or by a demethylation process. Furthermore, the apparent need for the rapid excision of $O^6$-methylguanine would lend support for the hypothesis that $O^6$-methylation is a potential mutagenic event (Loveless, 1969) against which living organisms appear to have evolved a protective mechanism. The excision rates for the bacterial system were made over a 1–3 hour period (Lawley and Orr, 1971) and although in hepatocytes the excision times are longer, nevertheless a half-life of about 13 hours for $O^6$-methylguanine is very rapid in relation to the life-span of rat hepatocytes. The cell renewal time for normal rat liver is reported as 400–450 days (MacDonald, 1961) and the cell cycle time has been estimated as 48 hours in the 8-week old rat (Post and Hoffman, 1964). Very few cells would be in cycle at any one time so that they could not play a significant part in determining the half-life times reported here.

*General considerations*

Single doses of DMN do not produce tumours in the liver of adult rats, whereas in the neonate a single dose given to mice (Toth, Magee and Shubik, 1964) or to rats (Della Porta and Terracini, 1969) produce a high incidence of tumours. On the other hand, exposure of adult rats to a chronic treatment (50 parts/$10^6$ in the diet; equivalent to about 4 mg/kg/day) does produce liver tumours (Magee and Barnes, 1956). Adult rats given this chronic exposure to the carcinogen showed an increased rate of turnover of liver DNA which was detected by the incorporation of [$^{14}$C]labelled adenine into the DNA of these animals (Craddock, 1971*a*). This evidence suggests that events during cell replication may be critical to the development of tumours and further support for this argument is provided by the observations of Craddock (1971*b*) that a single injection of the agent given to rats after partial hepatectomy, at times when DNA synthesis was taking place, resulted in the formation of tumours. Given that the excision of $O^6$-methylguanine proceeds relatively rapidly then a period of DNA synthesis would be essential before excision is completed, if the altered base is to have any effect, presumably through miscoding (Loveless, 1969). The same argument would hold for any other methylated base with a mutagenic potential.

In conclusion, the comparison made here between MMS and DMN treated animals suggests that if reaction with nucleic acids is germane to the process of liver carcinogenesis then those modifications to DNA structure most likely to be implicated are $O^6$-methylguanine and the unstable products yielding the early peak materials ($X_1$). Moreover, the detection of excision processes for abnormal components suggests that events leading to the development of tumours may be related to the efficiency of cellular excision systems for certain products of alkylation rather than to the level of alkylation obtained at a particular reaction site. Since $O^6$-methylguanine is chemically stable in DNA, it is possible that the presence of a rapid excision process for this base is important for maintaining the integrity of cellular DNA.

We are grateful to Mr P. F. Inman and Mr C. W. Woodruff for excellent technical assistance. This work was supported by grants to the Paterson Laboratories, Christie Hospital and Holt Radium Institute, by the Cancer Research Campaign and the Medical Research Council.

### REFERENCES

CAPPS, M. J. (1972) Interaction of the Carcinogen *N,N*-Dimethylnitrosamine with Rat Liver Nucleic Acids *in vivo:* Sites of Methylation and their Relative Stabilities. Ph.D. Thesis University of Manchester.

CRADDOCK, V. M. (1971*a*) Methylation of DNA in the Intact Animal and the Effect of the Carcinogens Dimethylnitrosamine and Ethionine. *Biochim. biophys. Acta*, **240**, 376.

CRADDOCK, V. M. (1971*b*) Liver Carcinomas Induced in Rats by Single Administration of Dimethylnitrosamine after Partial Hepatectomy. *J. natn. Cancer Inst.*, **47**, 899.

CRADDOCK, V. M. (1972) Analysis of DNA for the formation of 3-Methylthymine after administration of Dimethylnitrosamine. *Chem. Biol. Interactions*, **4**, 149.

CRADDOCK, V. M. & MAGEE, P. N. (1963) Reaction of the Carcinogen Dimethylnitrosamine with Nucleic Acids *in vivo*. *Biochem. J.*, **89**, 32.

DELLA PORTA, G. & TERRACINI, B. (1969) Chemical Carcinogenesis in Infant Animals. *Prog. exp. Tumor Res.*, **11**, 334.

DUTTON, A. & HEATH, D. F. (1956) The Preparation of $^{14}$C-Dimethylamine and $^{14}$C-Dimethylnitrosamine. *J. chem. Soc.*, 1892.

FRIEDMAN, O. M., MAHAPATRA, G. N., DASH, B. & STEVENSON, R. (1965) Studies on the Action of Diazomethane on Deoxyribonucleic Acid: The Action of Diazomethane on Deoxyribonucleosides. *Biochim. biophys. Acta*, **103**, 286.

KIRBY, K. S. & COOK, E. A. (1967) Isolation of Deoxyribonucleic Acid from Mammalian Tissues. *Biochem. J.*, **104**, 254.

LAWLEY, P. D. & BROOKES, P. (1963) Further Studies on the Alkylation of Nucleic Acids and their Constituent Nucleosides. *Biochem. J.*, **89**, 127.

LAWLEY, P. D. & ORR, D. J. (1970) Specific Excision of Methylation Products from DNA of *Escherichia coli* treated with *N*-Methyl-*N'*-nitro-*N*-nitrosoguanidine. *Chem. Biol. Interactions*, **2**, 154.

LAWLEY, P. D. & ORR, D. J. (1971) Reaction of Alkylating Mutagens with Nucleic Acids: N-3 of Guanine as a site of alkylation by *N*-Methyl-*N*-nitrosourea and Dimethyl Sulphate. *Chem. Biol. Interactions*, **4**, 431.

LAWLEY, P. D. & SHAH, S. A. (1972) Methylation of Ribonucleic Acid by the Carcinogens Dimethyl Sulphate, *N*-Methyl-*N*-nitrosourea and *N*-Methyl-*N'*-nitro-*N*-nitrosoguanidine: Comparisons of Chemical Analyses at the Nucleoside and Base Levels. *Biochem. J.*, **128**, 117.

LAWLEY, P. D. & THATCHER, C. J. (1970) Methylation of Deoxyribonucleic Acid in Cultured Mammalian Cells by *N*-Methyl-*N'*-nitro-*N*-nitrosoguanidine: The influence of Cellular Thiol Concentrations on the extent of Methylation and the 6-Oxygen Atom of Guanine as a Site of Methylation. *Biochem. J.*, **116**, 693.

LAWLEY, P. D., BROOKES, P., MAGEE, P. N., CRADDOCK, V. M. & SWANN, P. F. (1968) Methylated Bases in Liver Nucleic Acids from Rats treated with Dimethylnitrosoamine. *Biochim. biophys. Acta*, **157**, 646.

LOVELESS, A. (1969) Possible Relevance of 0–6 Alkylation of Deoxyguanosine to the Mutagenicity and Carcinogenicity of Nitrosamines and Nitrosamides. *Nature, Lond.*, **223**, 206.

LUDLUM, D. B. (1970) Alkylated Polycytidylic Acid Templates for RNA Polymerase. *Biochim. biophys. Acta*, **213**, 142.

LUDLUM, D. B. (1971) Methylated Polydeoxyribocytidylic Acid Templates for RNA Polymerase. *Biochim. biophys. Acta*, **247**, 412.

LUDLUM, D. B. & WILHELM, R. C. (1968) Ribonucleic Acid Polymerase Reactions with Methylated Polycytidylic Acid Templates. *J. biol. Chem.*, **243**, 2750.

MACDONALD, R. A. (1961) " Lifespan " of Liver Cells. *Archs intern. Med.*, **107**, 335.

MAGEE, P. N. & BARNES, J. M. (1956) The Production of Malignant Primary Hepatic Tumours in the Rat by Feeding Dimethylnitrosamine. *Br. J. Cancer*, **10**, 114.

MARGISON, G. P., CAPPS, M. J., O'CONNOR, P. J. & CRAIG, A. W. (1973) Loss of 7-Methylguanine from Rat Liver DNA after Methylation *in vivo* with Methly Methanesulphonate or Dimethylnitrosamine. *Chem. Biol. Interactions*. In press.

MCELHONE, M. J., O'CONNOR, P. J. & CRAIG, A. W. (1971) The Stability of Rat Liver Ribonucleic Acid *in vivo* after Methylation with Methyl Methanesulphonate or Dimethylnitrosamine. *Biochem. J.*, **125**, 821.

O'CONNOR, P. J., CAPPS, M. J., CRAIG, A. W., LAWLEY, P. D. & SHAH, S. A. (1972) Differences in the Patterns of Methylation in Rat Liver Ribosomal Ribonucleic Acid after Reaction *in vivo* with Methyl Methanesulphonate and *N,N*-Dimethylnitrosamine. *Biochem. J.*, **129**, 519.

POST, J. & HOFFMAN, J. (1964) Changes in the Replication Times and Patterns of the Liver Cell during the Life of the Rat. *Exp. cell. Res.*, **63**, 111.

SCHOENTAL, R. (1967) Methylation of Nucleic Acids by [$^{14}$C]-Methyl-*N*-nitrosourethane *in vitro* and *in vivo*. *Biochem. J.*, **102**, 5C.

SHOOTER, K. V. (1972) Some Aspects of the Interaction of Carcinogenic and Mutagenic Agents with Purines in Nucleic Acids. *Jerusalem Symp., Quant. Chem. Biochem.*, **4**, p. 509. Ed. E. Bergman and B. Pullman. Israel Academy of Sciences and Humanities.

SINGER, B. & FRAENKEL-CONRAT, H. (1969) Chemical Modification of Viral Ribonucleic Acid VIII. The Chemical and Biological Effects of Methylating Agents and Nitrosoguanidine on Tobacco Mosaic Virus. *Biochemistry*, **8**, 3266.

SINGER, B. & FRAENKEL-CONRAT, H. (1970) Messenger and Template Activities of Chemically Modified Polynucleotides. *Biochemistry*, **9**, 3694.

TOTH, B., MAGEE, P. N. & SHUBIK, P. (1964) Carcinogenesis Study with Dimethylnitrosamine Administered Orally to Adult and Subcutaneously to Newborn BALB/c mice. *Cancer Res.*, **24**, 1712.

WHITTLE, E. D. (1969) Methylation of Rat Liver RNA *in vivo* by Methyl Methanesulphonate. *Biochim. biophys. Acta*, **195**, 381.